# Factors associated with indication of tooth whitening during orthodontic treatment

**DOI:** 10.4317/jced.62472

**Published:** 2025-02-01

**Authors:** Sharon Chinchay-Ruesta, Mariano Ortiz-Pizarro

**Affiliations:** 1 Faculty of Medicine, Catholic University Santo Toribio de Mogrovejo, Chiclayo, Peru

## Abstract

**Background:**

Although teeth whitening is preferred after orthodontic treatment, aesthetic demands may generate a different indication. Aim: To determine the factors associated with indication of tooth whitening during orthodontic treatment.

**Material and Methods:**

A cross-sectional study was conducted using an online survey for participation of 200 dentists who frequently performed fixed orthodontic treatment. The validity and reliability of a 10 questions instrument was determined, content was entered into Survey Monkey software to generate delivery links. The instrument was sent through social networks with instant messaging and email. The bivariate analysis of associated factors was evaluated with Chi Square test and logistic regression was used to identify risk or protective factors for indication of tooth whitening.

**Results:**

In bivariate analysis, it was found that dentist’s specialization in orthodontics (*p*-value = 0.006), intensity of patient’s tooth staining (*p*-value = 0.001), patient’s staining habits (*p*-value = 0.001) and patient’s self-perceived aesthetic need (*p*-value = 0.001) were associated with indication of tooth whitening during orthodontic treatment. While professional experience (*p*-value = 0.525) and training in dental aesthetics (*p*-value = 0.936) were not associated. In adjusted analysis, differences were found between those who are specialists in orthodontics versus those who are not (*p*-value = 0.035), multiplying by 2.750 their decision to indicate tooth whitening (OR=2.750).

**Conclusions:**

The decision to indicate tooth whitening during orthodontic treatment is low. However, this initial decision could be affected by intensity of tooth staining, pigmentation habits and patient’s self-perceived need for dental aesthetics; but mainly by orthodontics specialization of dentist.

** Key words:**Tooth bleaching, fixed orthodontic appliance, brackets, dentists.

## Introduction

In recent years, aesthetics has gained considerable importance in dental practice due to social patterns that include ideal smile characteristics with respect to shape, size, position and color of teeth (1). Orthodontic treatment aims to solve problems of tooth position to restore not only function but also aesthetics (2). However, it has been observed that orthodontic appliances can contribute to tooth discoloration, as they increase retention of dental plaque and staining substances around of teeth, resulting in an unfavorable aesthetic effect (1,3).

In this context, it is common for patients to inquire about the possibility of improving dental aesthetics before, during or after orthodontic treatment (4). Although predominant recommendation is to postpone tooth whitening until active orthodontic treatment has finished (5), there are studies that suggest that patients continually seek aesthetic solutions. Even without professional advice, there is a high likelihood that whitening will be self-administered by the patient due to high demand for over-the-counter teeth whitening products, despite limited clinical evidence that exists on its benefits and risks it may entail (6,7).

Depending on professional’s indication, there are two techniques available that differ with respect to concentration of bleaching agent and exposure time to bleaching agent (8). In-office bleaching is a professionally supervised technique and therefore can safely use higher concentrations of hydrogen peroxide to achieve an immediate aesthetic result and rapid visual satisfaction in patient. Home bleaching generally requires cooperation of patient to use trays that will contain carbamide peroxide at low concentrations for a couple of weeks (5,6). Although it has been shown that in-office bleaching continues to produce better whitening effect, indication for home bleaching increased because it produces less tooth sensitivity, is less expensive, saves visits to office and fits better with patient’s lifestyle despite bleaching agent being supplied by professional (9). The trend towards patients being able to decide based on their social environment or commercial advertising of over-the-counter whitening products has further complicated situation for professionals who conceptually know that it is not ideal to perform tooth whitening during orthodontic treatment (10), but at same time recognize aesthetic desires of patient and must make decisions about an obvious color change that occurs during treatment (2,3).

Therefore, the aim of present study was to identify whether dentist factors such as professional experience, specialization in orthodontics and training in dental aesthetics; as well as patient factors such as intensity of tooth staining, pigmenting habits and self-perceived need for tooth aesthetics may be associated with indication of tooth whitening during fixed orthodontic treatment.

## Material and Methods

This cross-sectional study used an online survey and was approved by a research ethics committee through resolution No. 235-2024-USAT-FMED. The population consisted of 56,813 dentists registered in Peruvian Dental College until April 17, 2024.

Dentists who frequently performed fixed orthodontic treatment were included in study, while who could not be contacted via social media, who not respond to invitation or did not want to participate were excluded. The elimination criterion was established for dentists who initiated their participation but did not send answers to survey for any reason, in which case was replaced.

Sample size was calculated using Freeman formula: 10*(k+1), where value of K corresponds to number of independent or predictive variables (11). Considering frequency of indication for tooth whitening and six factors to be evaluated: professional experience, specialization in orthodontics, training in dental aesthetics, intensity of tooth staining, pigmentary habits and self-perceived need of patient; a minimum sample size of 70 dentists is obtained. Considering 50% response loss rate, an adjusted sample of 105 dentists was calculated. However, 200 dentists were finally included, with a test power reached of 0.916. Sampling was non-probabilistic by snowball, due to impossibility of obtaining a complete list with data from all participants.

The identification of professionals was carried out through social networks Facebook®, Instagram®, Twitter®, TikTok® and LinkedIn®; by reviewing public profiles that provided personal and professional data. Within recruitment of participants, dentist was registered, a public profile was implemented within each social network and a friend request was sent to generate trust that facilitates participation. Once request was accepted, the purpose of study was explained and selection criteria were verified. In case of accepting participation, a cell phone number with access to instant messaging applications and/or personal email was requested in order to send informed consent and survey. Where possible, each participant was asked to recommend the study to two other potential participants to improve recruitment.

The data collection instrument was entered into Survey Monkey® survey software to generate an electronic version of inventory used and coded submission links for each participant. The link from instrument was sent via instant messaging or email, and responses were either returned same day or delayed. For delayed responses, a first reminder was sent five days after link was sent, and two more attempts every seven days when necessary. The described methodology achieved an adherence rate of 87% with respect to total number of links sent.

Regarding instrument used, the content of an inventory of 10 closed questions was validated by a team of five experts and KR-20 test was used to determine a reliability of 0.891 within a pilot study. The instrument contains four sections: first section includes presentation of study, informed consent and general instructions; second section aimed at obtaining demographic data; third section includes questions about professional characteristics of dentist and clinical practice; fourth section includes questions related to patient characteristics.  Table 1 describes the questions and their response alternatives used in survey.

-Statistical analysis

The data provided by survey software were entered into Microsoft Excel v2019 spreadsheets (Microsoft, Redmond, WA, USA) for data curation. The data were exported to statistical program STATA v17 (StataCorp, College Station, TX, USA) for analysis. In univariate analysis, percentages and pie charts were used. In bivariate analysis, association of variables was individually evaluated using Pearson Chi Square test. In multivariate analysis, Backward Stepwise method was used to obtain a binomial logistic regression model to explain the indication for tooth whitening and included odds ratio (OR), beta coefficients and R2 of model; with a significance level of 5%.

## Results

**Figure 1:**
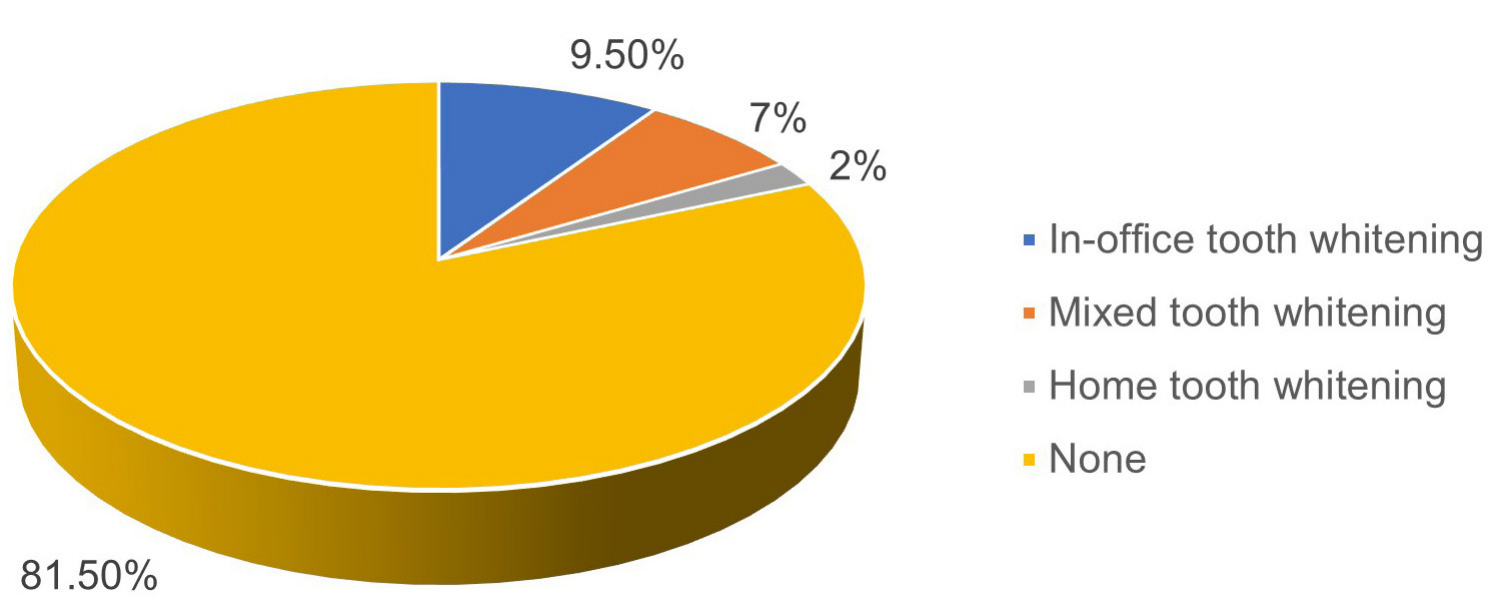
Indication of tooth whitening during fixed orthodontic treatment.

A total of 200 dentists participated in survey, over 40 years accounted for 52%, between 31 to 40 years for 41.5%, and up to 30 years for 6.5%. Regarding gender, 79% for men and 21% for women. Tooth whitening during fixed orthodontic treatment was indicated by 18.5% of total dentists participating in study, as shown in Figure 1. Within this percentage, 9.5% was an indication for in-office tooth whitening, 7.0% for mixed tooth whitening and 2.0% for home tooth whitening.

The indication for tooth whitening during orthodontic treatment may be associated with various professional characteristics and clinical practice of dentist, which are analyzed in  Table 2. Regarding professional experience, teeth whitening was indicated by 20% of dentists with more than 10 years of experience and by 16.5% of dentists with up to 10 years of experience; but it was not sufficient to establish an association between both variables (*p*=0.525). If dentist has a specialization in orthodontics, whitening is indicated by 26% of specialists; which reflects the association between specialization and indication for whitening (*p*=0.006). The dentist’s certified training in dental aesthetics was also evaluated. In this case, whitening was indicated by 18.4% of those who had training and by 18.9% of those who did not have it; no association was found (*p*=0.936).

This study demonstrated that patient characteristics may also affect initial decision on tooth whitening within a fixed orthodontic treatment as shown in  Table 3. In case of patients with intense tooth staining, 32.7% of dentists would indicate tooth whitening, while only 4.9% of dentists would indicate tooth whitening when stains were not intense; which determined a highly significant association between these variables (*p*=0.001). The pigmentation habits that patient shows or develops during treatment can influence change of an initial decision regarding indication of tooth whitening. In case the patients present these habits, whitening would be indicated by 31.4% of dentists and by 4.2% of them when this problem doesn’t exist, showing a highly significant association (*p*=0.001). Finally, there may be a need for tooth whitening that patient perceives regarding to improve oral aesthetics. When dentist takes this need of patient into consideration, there is an indication of tooth whitening of 30.2% while when this need is not expressed only 5.3% of dentists indicate tooth whitening within orthodontic treatment; showing again a highly significant association between variables (*p*=0.001).

The analysis of factors associated with dentist’s indication to indicate whitening or not, constituted a bivariate analysis with each factor. For this reason, an evaluation was performed using logistic regression to determine a multivariate analysis of each factor adjusted by effect of other factors. In  Table 4, the resulting model in a conservative adjustment (pseudo R2 = 0.305) indicates effects regarding option of not indicating tooth whitening within orthodontic treatment, because it was the most frequent decision of dentist. Differences were found between dentists who have a specialty in orthodontics versus those who are general dentists (*p*=0.035), specialists multiplied by 2,750 their decision not to indicate tooth whitening during orthodontic treatment compared to general dentists.

## Discussion

The present research aimed to evaluate the frequency of tooth whitening recommendations among dentists, mostly men and with a predominant age group over 40 years. In present study it was found that there is a low frequency of indication for tooth whitening, but this indication was susceptible to vary depending on various factors. This may be explained by potential insecurity associated with use of whitening agents on orthodontic appliances, a concern supported by studies reporting negative effects on optical properties, strength, and ion release (12-14). However, in recent studies the panorama improves when inflammatory parameters are evaluated (15), as well as whitening effect *in vitro* (16,17); although clinical performance of this effect is still debatable (15). Despite limited background, the frequency of recommendation or indication of whitening during orthodontic treatment can vary greatly compared to findings of present study. For example, Niño *et al*. (10) reported less than 5% among Colombian orthodontists, while Slack *et al*. (18) reported up to 76% among American orthodontists. These marked differences between the data provided by dentists in South America and North America could be explained by longer development history of American orthodontics and its earlier sociocultural adaptation (19).

Although majority of specialists and general dentists do not indicate tooth whitening during orthodontic treatment, it was found that dentists with a specialty in orthodontics indicate tooth whitening in more than double the percentage represented by general dentists. Even, according to results, a greater experience of the professional seems to partially reinforce this position, regardless of whether or not they have a background in dental esthetics. This could be explained by a more frequent exposure of orthodontists to immediate esthetic demands of adult patients seeking orthodontic treatment and where it is difficult to obtain satisfaction without complementing with other multidisciplinary esthetic treatments such as whitening (20,21).

After indication or not of tooth whitening during orthodontic treatment, it was found that this decision could be affected or modified according to intensity of tooth staining observed. This influence on the decision to indicate whitening could be due to a more technical and specific second evaluation by the professional, taking into account differences in shape, size, appearance of surrounding tissues, and type of orthodontic appliance; which also influence evaluation to intensity of color, allowing flexibility in decision to achieve patient satisfaction and maintain collaboration with treatment (20,22).

Staining habits are a factor to consider and can also open possibility of recommending tooth whitening during treatment. If verified that the patient has a history of a staining diet, present study found that this information would affect initial decision of whether or not to indicate whitening. According to scientific literature, there is no longer any doubt about staining ability of diet, not only on teeth but also on orthodontic appliances and attachments (5,23). However, effectiveness of using whitening toothpaste or mouthwash to control this everyday problem is still debaTable. This is due to non-uniform lightening effects achieved, alterations in surface of materials and decrease in their mechanical properties caused by various abrasive agents, which depend on concentration and commercial brand used (14).

It is also important to know if self-perceived need for dental esthetics that the patient communicates to dentist could affect an initial position on tooth whitening. In this study it was found that patient’s desire according to beliefs and perceptions about dental esthetics did influence this decision. In fact, dentists who frequently perform orthodontic treatment do recognize that their patients need teeth whitening as reported by Niño *et al*. (10) and Slack *et al*. (18); controversy revolves around indicating whitening at time of patient’s esthetic need or persuade the patient to wait until the end of treatment, which evidence suggests is better approach (5,14,24). This becomes more problematic considering the increasing frequency of orthodontic treatments in adults among a predominantly commercial society (21). Many adults who previously perceived themselves naturally as orthodontic patients now adopt the role of aesthetic consumers and self-identify as such (25). Additionally, it has been reported that not only patients may push for cosmetic service of teeth whitening, but also dentists motivated by other socioeconomic and geographic variables (26).

Evaluating these reported influences together, it can be confirmed that the dentist’s specialization in orthodontic treatment is a cornerstone for managing clinical decisions (27). Thus, specialization was identified as a factor that nearly triples the likelihood of not recommending whitening during treatment, which could give an idea of an important but not absolute influence compared to other variables that have not yet been studied. Specialization is a characteristic that would be related to better probabilities of adequate multidisciplinary care within a treatment undeniably from esthetic nature (26), also with better options for review of specific scientific literature and more casuistry where there is a greater possibility of observing adverse effects of whitening within orthodontic treatment (14,27).

This study collected valuable information on the clinical decision whether or not to indicate tooth whitening during orthodontic treatment. Although valid and reliable documentary instruments were used, among limitations we can mention that due to diversity of clinical protocols and factors involved in a decision, answers collected do not necessarily reflect a real decision of professionals on supposed situations. In this sense, brevity of survey allowed a high response rate compared to other similar studies that had this aspect as a limitation. However, these results were obtained from a non-representative sample, so it is not possible to determine whether this aspect could have affected the answers provided by dentists.

## Conclusions

Dentists in general indicated tooth whitening during orthodontic treatment with low frequency, but it was twice as frequent in orthodontic specialists as in general dentists, and no association was found with professional experience or with any training in dental esthetics. This initial indication for bleaching could later be changed if dentists take into account the intensity of possible tooth staining, staining habits or the patient’s self-perceived need for esthetics. Considering all the variables evaluated, specialization in orthodontics is a significant factor that protects against the decision to indicate tooth whitening during orthodontic treatment; however, this variable would be insufficient to explain a decision that is frequently made in orthodontic clinical practice.

## Figures and Tables

**Table 1:** Inventory type instrument used in survey.

Question	Response
Demographic data	
Sex	Male Female
Age	____ years
Professional characteristics and clinical practice	
How many years of experience do you have performing fixed orthodontic treatments?	Up to 10 years More 10 years
Regarding clinical practice in orthodontics, do you perform orthodontic treatments as orthodontist or general dentist?	General dentist Orthodontist
Do you have any certified training in dental aesthetics (course, diploma or specialty)?	Yes No
Do you usually indicate tooth whitening during fixed orthodontic treatment?	Yes No
Patient characteristics	
Considering answer to previous question, could intensity of the patient's tooth staining change your decision about indication of tooth whitening within fixed orthodontic treatment?	Yes No
Considering answer to previous question, could the patient's staining habits change your decision about indication of tooth whitening within fixed orthodontic treatment?	Yes No
Considering answer to previous question, could the patient's self-perceived need for dental aesthetics change your decision about indication of tooth whitening within fixed orthodontic treatment?	Yes No

**Table 2:** Characteristics of dentist and association with indication for tooth whitening during orthodontic treatment.

Variable	Indication for tooth whitening		X^2^	*p-value
	Yes n=37	No n=163		
Professional experience in fixed orthodontic treatments				
Up to 10 years	14(16.5)	71(83.5)	0.404	0.525
More 10 years	23(20.0)	92(80.0)		
Clinical practice in orthodontics				
Orthodontist	26(26.0)	74(74.0)	7.461	0.006*
General dentist	11(11.0)	89(89.0)		
Certified training in dental aesthetics				
Yes	27(18.4)	120(81.6)	0.006	0.936
No	10(18.9)	43(81.1)		

Categorical data expressed as absolute numbers and percentages (%). *p-value: significance level ≤ 0.05. X2: Chi-square test.

**Table 3:** Patient characteristics and association with indication for tooth whitening during orthodontic treatment.

Variable	Indication for tooth whitening		X^2^	*p-value
	Yes n=37	No n=163		
Intensity of tooth staining				
Yes	32(32.7)	66(67.3)	25.529	0.001*
No	5(4.9)	97(95.1)		
Staining habits				
Yes	33(31.4)	72(68.6)	24.506	0.001*
No	4(4.2)	91(95.8)		
Self-perceived need for dental aesthetics				
Yes	32(30.2)	74(69.8)	20.437	0.001*
No	5(5.3)	89(94.7)		

Categorical data expressed as absolute numbers and percentages (%). *p-value: significance level ≤ 0.05. X2: Chi-square test.

**Table 4:** Factors associated with indication of tooth whitening during orthodontic treatment – Logistic regression model.

Variable	B	SE	Wald	*p-value	OR	Cox & Snell R^2^	Nagelkerke R^2^
Age, 20-30	0.026	0.989	0.001	0.979	1.026	0.188	0.305
31-40	-0.712	0.569	1.562	0.211	0.491		
Sex, male	-0.486	0.495	0.964	0.326	0.615		
Professional experience, up to 10 years	0.346	0.607	0.324	0.569	1.413		
Orthodontist	1.011	0.481	4.429	0.035*	2.750		
Certified training in dental aesthetics	0.014	0.471	0.001	0.976	1.014		
Intensity of tooth staining	1.342	0.843	2.534	0.111	3.828		
Staining habits	0.737	0.898	0.672	0.412	2.089		
Self-perceived need for dental aesthetics	0.778	0.670	1.349	0.245	2.177		
Constante	-3.521	0.949	13.771	0.000	0.030		

*p-value: significance level ≤ 0.05.

## Data Availability

The datasets used and/or analyzed during the current study are available from the corresponding author.
